# Phorbol ester degradation in Jatropha seedcake using white rot fungi

**DOI:** 10.1007/s13205-013-0174-9

**Published:** 2013-09-26

**Authors:** Anjali Bose, Haresh Keharia

**Affiliations:** BRD School of Biosciences, Sardar Patel Maidan, Sardar Patel University, Satellite Campus, Vadtal Road, P.O. Box 39, Vallabh Vidyanagar, 388 120 Gujarat India

**Keywords:** Jatropha seedcake, White rot fungi, Phorbol ester

## Abstract

White rot fungi are well known for their ability to degrade a wide range of xenobiotics due to their enzymatic systems. Therefore, the present investigation was aimed at screening ten different white rot fungi for degradation of phorbol esters from Jatropha seedcake (JSC). The JSC was fermented with pure cultures of white rot fungi for 20 days under solid state condition. All the white rot fungi tested exhibited degradation of phorbol ester during fermentation of JSC without adversely influencing the nutritional properties of the seedcake. *Ganoderma lucidum* and *Trametes zonata* were found to degrade phorbol ester in JSC to undetectable levels. This study demonstrates the potential of white rot fungi for degradation of phorbol esters, a major anti-nutritional factor, in JSC preventing its utilization as cattle feed.

**Electronic supplementary material:**

The online version of this article (doi:10.1007/s13205-013-0174-9) contains supplementary material, which is available to authorized users.

The bio-diesel production from *Jatropha curcas* L. gained momentum due to its inedible oil content that can be converted to fuel without competing with the food market. The extraction of oil from Jatropha seeds is associated with generation of substantial amount of seedcake waste at an average rate of 500 g cake per kg of seeds used (Zanzi et al. 2008). Inspite of its high protein content along with presence of all essential amino acids, except lysine (Makkar and Becker 1997b), it cannot be used in feed formulation due to the presence of potential anti-nutritional components like phorbol esters (PE), lectins and trypsin inhibitors (Makkar et al. 1997). The PEs, have been identified as main toxicants in JSC, which could not be destroyed even by heating at 160 °C for 30 min (Makkar et al. 1998) and, therefore, its removal is currently an important issue to be addressed. Several physico-chemical methods have been developed for PE removal, but none has proved to be economically feasible (Aregheore et al. 2003; Martínez-Herrera et al. 2006; Rakshit et al. 2008).

In this context, use of JSC as substrates for microbial fermentation would not only add to its utility, but during the process of fermentation there exists a possibility of degradation of anti-nutritional factors present in it, thereby solving its subsequent disposal issues. The feasibility of this approach has been demonstrated for reducing gossypol in cotton seed meal using *Geotrichum candidum* (Sun et al. 2008) and ricin in castor seedcake by *Paecilomyces variotii* (Madeira et al. 2011).

As white rot fungi are well known for their ability to degrade a wide range of xenobiotics, such as polyphenolic compounds and synthetic dyes, due to the secretion of extracellular enzymes (Asgher et al. 2008; Alberts et al. 2009), the present study was undertaken to investigate the ability of ten different white rot fungi, namely, *Ganoderma lucidum* (GL), *Pleurotus florida* (PF), *Pleurotus sapidus* (PS), *Pleurotus sajor*-*caju* (PSC), *Pleurotus ostreatus* (PO), *Phanerochaete chrysosporium* (PC), *Trametes hirsute* (TH), *Trametes zonata* (TZ), *Trametes gibbosa* (TG) and *Trametes versicolor* (TV) for degradation of PE in deoiled JSC. They were grown on 2 % (w/v) malt extract agar plates at 27 °C, preserved at 4 °C on malt extract agar slopes and maintained by subculturing once in 2 months.

Twenty-five grams of JSC (obtained from Food Processing and Bioenergy division, Anand Agriculture University, Gujarat) was taken in 250-mL Erlenmeyer flask, moistened with 30 mL of distilled water and autoclaved. The flasks were inoculated with two blocks (1 cm × 1 cm) of actively growing individual fungal culture, followed by incubation at 30 °C and 70 % relative humidity. An uninoculated flask served as experimental control. After 20 days of fermentation, the content of the flasks were extracted and analyzed for PE and nutrients. All the experiments were done in triplicates.

Phorbol esters were extracted by following the method described by Joshi et al. (2011) and quantified using C-18 reverse-phase HPLC employing a Luna 18 column (250 × 4.6 mm, octadecyl group, particle size 5 μm) procured from Phenomenex (USA). The separation was carried out with the solvent system: water and acetonitrile (40 % acetonitrile for 15 min followed by gradient of 40–75 % acetonitrile for 20 min and then to 100 % acetonitrile for 5 min and finally returned to 40 % for the next 5 min) with a flow rate 1.3 mL/min at 25 °C. The detector (Photo Diode Array) wavelength was set on 280 nm and 5 μL of sample was injected for analysis.

Normally, *Jatropha* sp. is reported to have four to six PEs or its derivatives (Haas et al. 2002; Barros et al. 2011), out of which, phorbol-12-myristate 13-acetate (PMA) is the major PE in *J. curcas* (Makkar and Becker 1997a). However, we observed three major peaks at 25.1, 25.26 and 25.6 min (Fig. 1a), which appeared at a lower retention time as compared to external standard PMA (Sigma Chemical Co., USA) dissolved in absolute methanol that appeared at 33.09 min. The type and quantity of individual PEs in Jatropha seed depends on the genotype of plant and the prevailing soil/climatic conditions (Martínez-Herrera et al. 2006). Makkar et al. (1997) reported varying concentration of PE from 0.8 to 3.3 mg/g in *J. curcas* kernel meal from different geographical sites. Ahmed and Salimon (2009) analyzed three different varieties of tropical *J. curcas* from Malaysia, Indonesia and India and observed two, five and four PE peaks, respectively.

**Fig. 1:**
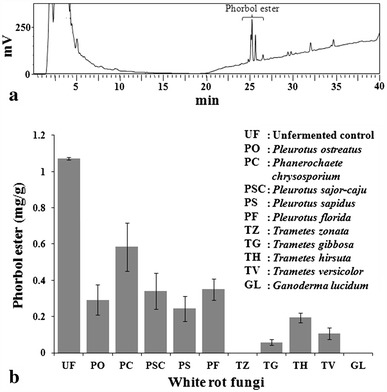
**a** HPLC chromatogram of phorbol esters from unfermented Jatropha seedcake and **b** Effect of fungal treatment on phorbol ester concentrations

The fermentation of JSC with edible white rot fungi lowered PEs content from 1.072 mg/g in unfermented control up to undetectable level in fermented seedcake (Fig. 1 and supplementary figure). The degradation of PE was observed by all the white rot fungi tested but the extent of degradation varied with the fungal culture (Fig. 1b). *G. lucidum* and *T. zonata* were found to completely degrade PEs in JSC. Trace amounts of PEs could be detected in JSC upon fermentation by *T. versicolor* and *T. gibbosa*, whereas *Pleurotus* sp. degraded ~70 % of initial PE present. However, *Ph. chrysosporium* could only reduce PE content up to ~45 %. The extent of reduction in PE content by *G. lucidum*, *T. zonata*, *T. versicolor* and *T. gibbosa* was comparable to the reports by Barros et al. (2011) and Belewu and Sam (2010).

Further the nutritive value was evaluated using the protocols of Indrayan et al. (2005) with few modifications. The analysis involved the determination of moisture content by drying samples at 105 °C to constant weight. Ash content and the total organic matter were estimated by determining the loss in weight after igniting the samples in a muffle furnace at 550 °C for 12 h. Dry fermented seedcake was digested using concentrated H_2_SO_4_ followed by distillation with NaOH in Kel Plus Nitrogen estimation system (Classic DX, Pelican Equipments) prior to determination of total nitrogen. Total nitrogen was estimated through titration of the distillate collected from the Kel Plus distillation unit against 0.1 (N) H_2_SO_4_. The total protein content was determined by multiplying total Kjeldahl nitrogen with 6.25. Total fat was determined by extracting 2 g sample with hexane in a Soxhlet extractor for 6 h. The hexane extract was then evaporated and residue left was weighed to determine total fat. The total carbohydrate and nutritive value was then calculated using following equations:

1 $$\text{Carbohydrate}\;\left(\%\right)\;\text{was given by:}\;100-\left(\text{Ash}\;\%\;+\;\text{Fat}\;\%\;+\;\text{Protein}\;\%\right).$$

2 Nutritive value was determined by:4×Protein%+9×Fat%+4×Carbohydrate%.

To determine total *P* and *K*, the samples were first digested with a mixture of nitric acid and perchloric acid (9:4). Phosphorus in the digestate was estimated through formation of vanado-molybdo-phosphoric–heteropoly complex followed by absorbance measurement at 420 nm (Gupta 2004). The potassium was measured in the digestate using flame photometer (Singh et al. 2007).

The results of proximate composition of fungal fermented and unfermented JSC (Table 1) exhibited slight increase in protein content (2.03–6.92 %). The increment in the protein content could be due to the addition of microbial protein during the process of fermentation. Ash content was found to increase significantly in JSC upon fermentation by all the white rot fungi tested. The increase in ash content of fermented seedcake may be considered as an indicator of mineralization (Table 1). Similar observation regarding increase in ash content has been reported for cotton waste upon fermentation by *Volvariella volvacea* (Akinyele and Akinyosoye 2005). Further, the fat content remained almost unchanged in most of the treatments, however, 4–9 % decrease in carbohydrate content was observed. Such decrease in carbohydrate content may be attributed to its utilization as nutrient source during fungal colonization. In present study, the overall nutritive value of the fermented seedcake remained almost unchanged. However, increase in nutritive value of agrowastes upon fermentation by fungi has been reported by Akinyele and Akinyosoye (2005).

**Table 1:** Nutritive composition of unfermented (control) and fermented Jatropha seedcake

Treatments	Protein (%)	Fat (%)	Ash (%)	*P* (ppm)	*K* (ppm)	Moisture (%)	Carbohydrate (%)	Nutritive value
UF	23.33 ± 0.48	0.65	5.83 ± 0.17	56	3,250	58.3 ± 0.28	11.99	146.73
PO	25.92 ± 0.06	0.7	7.55 ± 0.16	189	4,830	55.7 ± 0.41	10.13	150.5
PC	25.26 ± 0.25	0.65	7.89 ± 0.31	175	6,350	51.57 ± 0.22	14.63	165.41
PSC	26.44 ± 0.3	0.7	7.64 ± 0.18	168	6,650	53.95 ± 0.48	11.27	157.14
PS	30.15 ± 0.39	0.65	7.47 ± 0.01	189	6,750	54.73 ± 0.75	7	154.45
PF	25.33 ± 0.25	0.7	7.63 ± 0.58	189	4,730	53.45 ± 0.15	12.89	159.18
TZ	27.33 ± 0.12	0.6	6.98 ± 0.3	182	6,300	56.65 ± 0.19	8.44	148.48
TG	28.05 ± 0.15	0.5	7.43 ± 0	161	5,230	55.48 ± 0.31	8.54	150.86
TH	29.46 ± 0.63	0.55	7.28 ± 0.16	175	2,550	53.64 ± 0.43	9.07	159.07
TV	29.39 ± 0.07	0.55	7.74 ± 0.2	175	5,830	52.44 ± 0.72	9.88	162.03
GL	28.07 ± 0.12	0.5	8.24 ± 0.18	175	6,680	54.86 ± 0.05	8.33	150.1

*UF* unfermented Jatropha seedcake as experimental control, *PO* *Pleurotus ostreatus*, *PC* *Phanerochaete chrysosporium*, *PSC* *Pleurotus sajor-caju*, *PS* *Pleurotus sapidus*, *PF* *Pleurotus florida*, *TZ* *Trametes zonata*, *TG* *Trametes gibbosa*, *TH* *Trametes hirsuta*, *TV* *Trametes versicolor* and *GL* *Ganoderma lucidum* fermented Jatropha seedcake

Thus, the present investigation clearly demonstrated that solid state fermentation of JSC by white rot fungi could totally remove PE content and could be applied for large scale detoxification. Apart from this, the fermented seedcake would then retain high protein content and other nutritional values applicable to the animal feed industry.

## Electronic supplementary material

Below is the link to the electronic supplementary material.

Supplementary material 1 (DOC 152 kb)
