# Supplementary material for: Phorbol ester degradation in Jatropha seedcake using white rot fungi
Source: 3 Biotech. 2013 Sep 26;4(4):447–50. doi: 10.1007/s13205-013-0174-9 (PMC4145619; doi:10.1007/s13205-013-0174-9)

**Supplementary Figure 1:** HPLC chromatogram of phorbol esters from unfermented (control) JSC (a) and JSC fermented with (b) *P. ostreatus*, (c) *Ph. chrososporium*, (d) *P. sajor-caju*, (e) *P. sapidus*, (f) *P. florida*, (g) *T. zonata*, (h) *T. gibbosa*, (i) *T. hirsuta*, (j) *T. versicolor* and (k) *G. lucidum*.


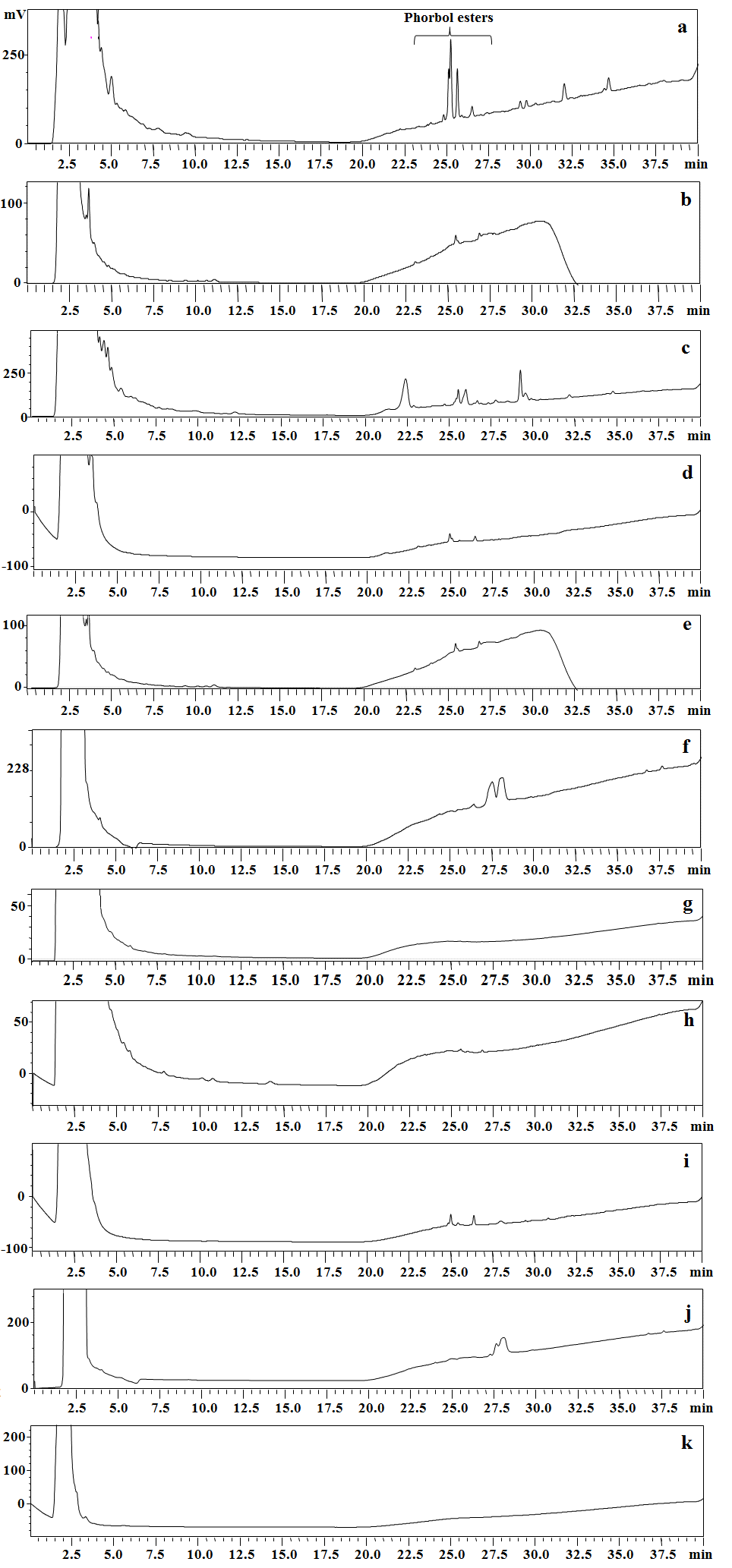

Supplement: Supplementary file 1 — Supplementary material 1 (DOC 152 kb) [file 13205_2013_174_MOESM1_ESM.doc]
